# Addition of *Trans*-Resveratrol-Loaded Highly Concentrated Double Emulsion to Yoghurts: Effect on Physicochemical Properties

**DOI:** 10.3390/ijms23010085

**Published:** 2021-12-22

**Authors:** Rocío Díaz-Ruiz, Amanda Laca, Marta Sánchez, Manuel Ramón Fernández, María Matos, Gemma Gutiérrez

**Affiliations:** 1 Department of Chemical and Environmental Engineering, University of Oviedo, Julián Clavería 8, 33006 Oviedo, Spain; uo219049@uniovi.es (R.D.-R.); lacaamanda@uniovi.es (A.L.); marta.ssotero@gmail.com (M.S.); UO245369@uniovi.es (M.R.F.); matosmaria@uniovi.es (M.M.); 2 Instituto Universitario de Biotecnología de Asturias, University of Oviedo, 33006 Oviedo, Spain

**Keywords:** *trans*-resveratrol, encapsulation, high internal phase double emulsions, yoghurt

## Abstract

*Trans*-resveratrol (RSV) needs to be encapsulated to maintain its beneficial properties on the human body. This is due to its extreme photosensitivity, short biological half-life, and easy oxidation. In this study, the use of double emulsions for RSV encapsulation and their further application on functional yoghurts was studied. Different types of yoghurts were prepared: with and without RSV and with two types of volumetric emulsion formulations (20/80 and 30/70). In order to study the influence of the addition of double emulsions to the physical properties of the prepared yoghurts, they were characterised fresh and after a month under storage at 4 °C, in terms of droplet size, morphology, stability, rheology, texturometry, colorimetry, and antioxidant capacity. Results obtained showed that the presence of emulsion in the yoghurts produced a generalised decrease in the predominant droplet size (from 48 µm to 15–25 µm) and an increase in the stability. Additionally, a predominantly elastic character was observed. The firmness values obtained were very similar for all the yoghurts analysed and did not suffer important modifications with time. A slight colour variation was observed with storage time in the control sample, whereas a more notable variation in the case of emulsion yoghurts was observed. An appreciable increase of the antioxidant capacity of the final functional yoghurt (100 g) was observed when it contained 5–8 mg of RSV. Encapsulated RSV added to yoghurts presented a larger protection against RSV oxidation compared with free RSV, presenting a larger antioxidant inhibition after one month of storage. Moreover, the antioxidant capacity of yoghurts with encapsulated RSV was not affected under storage, since slight reductions (3%) were registered after one month of storage at 4 °C.

## 1. Introduction

The preparation and consumption of functional foods have been growing in the last decades [1,2]. Functional foods are those foods that in addition to providing basic nutrients, have certain characteristics that provide other additional beneficial effects to the health of the consumers due to the ingestion of biocompounds incorporated in them [3,4]. 

The introduction of antioxidant compounds on a food matrix by its encapsulation in colloidal systems has been shown to be a suitable technique for antioxidant compounds administration, since it enhances the antioxidant power, reduces the degree of cellular oxidation and, therefore, prevents the appearance of different diseases [3]. In addition, antioxidant compounds have been proved to reduce dyslipidemias [5], decrease inflammatory processes [6], and prevent cardiovascular [7], kidney and nerve diseases [4].

Some authors [8] summarised the beneficial effects of RSV in clinical trials conducted in human patients with type 2 diabetes, obesity, cardiovascular disease, cancer, or skin disorders [9]. By relating the oxidative stress with an inflammatory process, it was found that dysbiosis could be involved in different disease development (obesity, diabetes, for instance). Dysbiosis is favoured by diet, age, and genetic variability, and affects the progression of microbiome-linked diseases; diverse phenolic compounds, such as RSV, employed as nutraceuticals have demonstrated they are capable of improving the metabolic balance of gut microbiota [10,11].

The benefits of taking RSV have also been tested in healthy patients; for example, it has been shown on 41 subjects that RSV protected against atherosclerosis [12]. Moreover, the biocompound is able to decrease fasting glucose levels and bilirubin levels [13] or decrease levels of C-reactive protein and triglycerides, and increase total antioxidant levels [6]. Another proven benefit of RSV is how RSV dose-dependently increased cerebral blood flow [14].

Within the great diversity of products offered as functional foods, there are products enriched with antioxidants, a wide family of compounds among which are carotenoids, ascorbic acid or polyphenols, a group within which RSV is included. In this context, it should be mentioned that many pathologies are closely associated with the imbalance of the microbiota of the human colon and polyphenolic nutraceuticals have been reported as potential compounds to reduce the oxidative stress by acting directly on the microbiota and combating the inflammation [11]. This trend has prompted the food industry to seek new technological solutions; as a result, the global market for functional foods has grown significantly in recent years [2,15].

The naturally polyphenol *trans*-resveratrol (3, 5, 4′-trihydroxystilbene) has an antioxidant nature that produces beneficial effects against several diseases, such as, for example, cancer, diabetes, neurodegeneration, cardiovascular disorders, inflammation, and other age-related pathologies [8,16,17,18,19,20,21]. This aspect has caused a notable increase in the use of this polyphenol in the cosmetic and pharmaceutical industries [22,23].

However, the problem of this compound is related to its photosensitive character and low stability [24,25]. To avoid this, the resveratrol can be encapsulated using different types of colloidal systems, such as vesicles [15,26,27,28], nanoemulsions [29] or water-in-oil-in-water (W_1_/O/W_2_) double emulsions [30,31,32,33], among others. Currently, the use of this compound in various basic foods, such as yoghurts, is being investigated with the aim of obtaining new functional foods enriched with resveratrol [24,25,31].

Encapsulation technology was defined by several authors [34] as a promising approach that has been employed for the protection and controlled release of different bioactive compounds including natural antioxidants. However, due to some intrinsic characteristics, e.g., low solubility, short shelf life, difficultly in their packaging and handling, losses due to environmental stresses and food processes, undesirable flavours and odours, untargeted release, and instability in various conditions during digestion in the gastro-intestinal tract, the application of these bioactive compounds in real food products, pharmaceuticals, and cosmetics has been limited. The use of double emulsions can overcome these restrictions [35].

Because of their complex structure, multiple emulsions can be viewed as systems that control the transport of molecules from an external to an internal phase or vice versa. Therefore, a common use of double emulsions is as biocompounds carriers [32,36,37,38,39,40,41,42,43,44,45] in the health sector (pharmaceutical and medical) and also in the cosmetic and food industry. Its application in these fields is related to their feasibility as drug delivery systems, since they protect short half-life compounds and administer them repeatedly by ingestion or injection [46,47]. The single administration of the active component through a double emulsion has shown prolonged-release properties, avoiding alterations caused by the environment (e.g., oxidation, light, enzymatic degradation) or during food digestion [48,49].

The benefits of using this type of emulsion for the administration of active compounds are to avoid alterations caused by the environment, such as oxidation or photodegradation, or those caused by the gastric juices during digestion [48,49]. Hydrophilic bioactive compounds, such as resveratrol, are encapsulated in the droplets of the inner phase, while the drops of the intermediate phase, acting as a barrier, protect the compound from environmental factors improving the stability and increasing the shelf life of the compound. However, the use of double emulsions has been restricted by the fact that they are unstable thermodynamic systems due to an excess of free energy associated with the emulsion droplets surface [49,50]. To formulate a W_1_/O/W_2_ double emulsion, at least two stabilisers are introduced into the system: a lipophilic one, to form the primary W_1_/O emulsion, and another hydrophobic stabiliser as a secondary emulsifier, to form the final multiple emulsion, which makes this system not always stable enough for biocompounds encapsulation.

It is important to point out that functional foods have a better acceptance by consumers compared to traditional tablets or pills, which is directly related to the ingestion of medicaments.

Moreover, some recent publications also indicate that functional foods consumption has notably increased in the last decade [51]. Specifically, an increase of the functional food market by up to 7.8% from 2018 to 2023 is expected. However, some problems associated with functional foods consumption related to the lack of knowledge and poor transparency of labels are emerging, which is an indication of the need to legislate on this matter [1,52].

In the present work, a new functional food consisting of yoghurt enriched with resveratrol was prepared. For this purpose, two different ratios of double emulsions were used as vehicles for encapsulation. The main goal of the present work was to study the effect produced by the presence of a double emulsion on the physicochemical properties of the final yoghurt, since these properties are related to the fabrication process and consumers’ acceptance.

The droplet size and morphology of the emulsions formulated were measured. Once the emulsions were introduced into the yoghurt, the samples were characterised in terms of droplet size, stability, morphology, colorimetry, rheology, and texturometry. Moreover, the antioxidant capacity of the prepared yoghurts was examined. All the analyses were carried out in fresh samples and after one month of storage at 4 °C (in the absence of light).

## 2. Results and Discussion

The five types of yoghurts elaborated were analysed the day after their elaboration (fresh) and after 1 month stored at 4 °C (yoghurts month). This way, the influence of adding different types of emulsion (double emulsions formulated with simple emulsions of ratios 20/80 and 30/70 of W_1_/O) with encapsulated RSV to the yoghurts was studied.

The use of the two W_1_/O volumetric ratios of 20/80 and 30/70 for the double emulsion formulations added to the yoghurts were selected according to previous results obtained, where it was stated that these were the highest internal phase ratios that could be achieved for a double W_1_O/W_2_ emulsions preparation without scarifying the emulsion stability [53].

Furthermore, the emulsions prepared were characterised in order to be able to evaluate their possible effect on the behaviour of the yoghurt.

### 2.1. Droplet Size Distribution

The droplet size distribution of the yoghurts was measured with and without double emulsions with 20/80 and 30/70 of volumetric internal ratios. In both cases, the emulsions were added with or without RSV.

**Figure 1:**
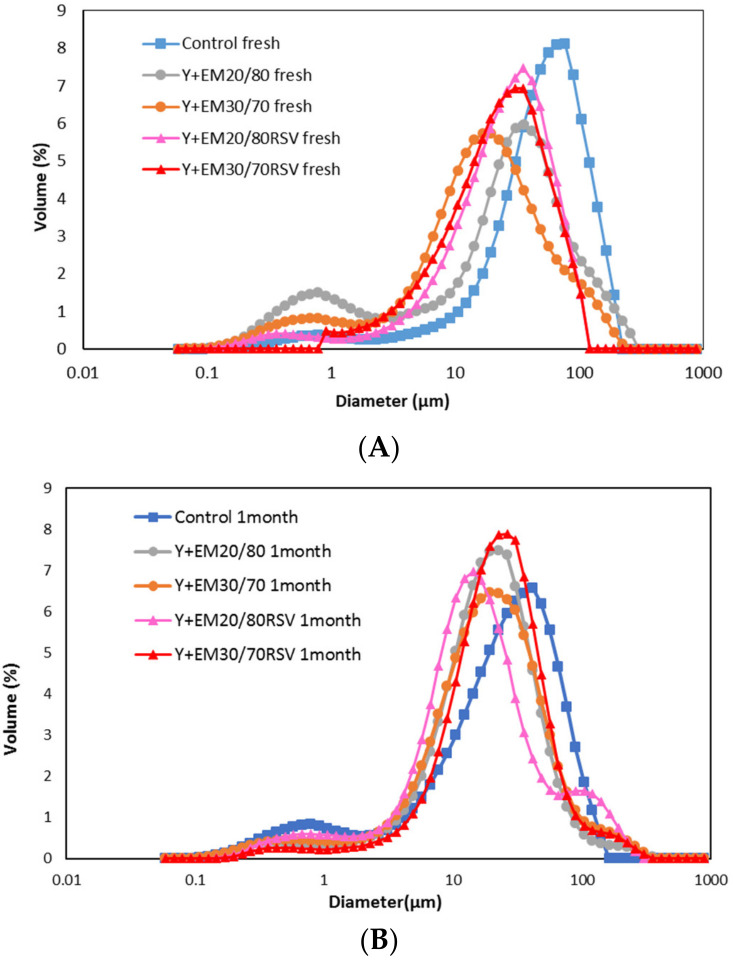
Droplet size distribution of the control yoghurt and of the yoghurts with double emulsions formulated by varying the volumetric ratio of the dispersed phase (primary emulsion W_1_/O) in relation to the external aqueous phase (W_2_), fresh (**A**) or after storing 1 month at 4 °C (**B**).

The droplet size distributions of the different types of fresh yoghurts prepared are shown Figure 1A, while the mean diameter and D_[4,3]_ are presented in Table S1 (Supplementary Materials).

All yoghurts prepared present a clear wide distribution, which indicates that the mean value will not always give an appropriate indication of the representative diameter of the sample. It can be seen that in all the samples the presence of the emulsion affects the size distribution of the yoghurt with a smaller mean drop size than the control yoghurt. This change in droplet size can be attributed to the presence of oil droplets in the emulsion itself, since emulsions usually have a smaller droplet size than the ones measured in the yoghurt in the present work [33,53].

For yoghurts with emulsions without RSV, a bimodal distribution was observed, a secondary peak around 0.5 µm could be appreciable even though its volume is close to 10 times lower than the volume of the principal peak located around 10–100 µm. This secondary small peak has been observed in previous works where double emulsions were prepared and characterised [31]. However, the presence of this secondary peak is nearly not noticeable when RSV is present in the emulsions added to the yoghurt. This indicates that the presence of RSV has some interactions with the food matrix, indicating that it is partially located at the oil/water interface, as in previous works, which observed that emulsions with RSV presented a larger stability than those without RSV, due to the additional stabilising character of RSV that can be located at the oil/water interface [29]. In the present work, double emulsions were added to the yoghurt. Due to the aqueous character of the external phase of the yoghurt, the external aqueous phase of the added double emulsion was completely homogenized and mixed with the yoghurt matrix, while the W_1_/O droplets suspended on the food matrix remained. Proteins are well known to be adequate emulsion stabilisers, [54,55] and hence the presence of RSV at the W_1_/O interface could favour the protein penetration through the oil drop producing a larger interaction between the emulsion and the yoghurt compounds.

However, for samples one month old, no notable differences were observed when different types of emulsions were incorporated in the yoghurt.

Looking at Figure S1, where yoghurts particle size distributions are individually compared between fresh and one-month-old sample, it can be observed that the mean droplet size has been reduced for all samples, especially for those of plain yoghurt and those with an emulsion of ratio 20/80; after one month of storage, the effect produced by the incorporation of an emulsion in the yoghurt is minimal, getting in all cases mean droplets values similar to those obtained for plain yoghurts, as can be easily compared in Table S1.

### 2.2. Colloidal Stability

**Figure 2:**
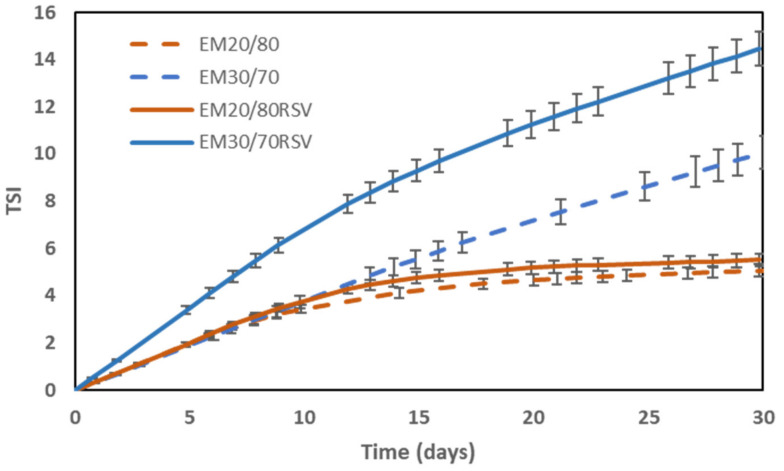
Evolution of the Turbiscan Stability Index (TSI) of the different types of emulsions over time to study how the addition of RSV to emulsion affects stability.

**Figure 3:**
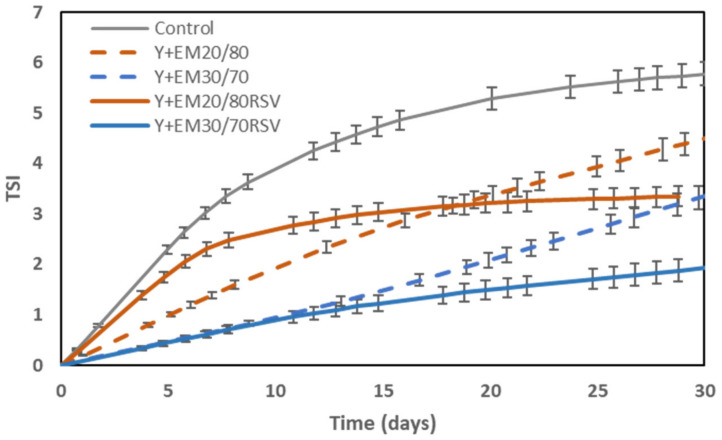
Evolution of the Turbiscan Stability Index (TSI) of the different types of yoghurts over time to study how the addition of emulsion to yoghurt affects stability.

The stability results obtained for the emulsions formulated (Figure 2) and for the yoghurts prepared during one month of storage at 4 °C (Figure 3) are shown below.

In Figure 2, it can be seen that the emulsions with a 20/80 internal phase ratio present a greater stability than those with a 30/70 internal phase ratio, since TSI values are smaller during the 30 days of storage. Large differences can be observed after 15 days of storage. On the other hand, the presence of RSV in the emulsion does not seem to have any influence on the emulsion’s stability for emulsions with an internal ratio of 20/80. However, for emulsions with an internal ratio of 30/70, the presence of RSV negatively affects the stability of the emulsion, because the sample with RSV presents higher TSI values from the very first days of storage. Previous works have showed the negative effect of a large internal emulsion ratio on double emulsion’s stability [33], for example, in previous studies [53] where the concentration of the inner emulsion (W_1_/O) for several external (W_1_O/W_2_) ratios was optimized in terms of encapsulation efficiency (EE), colloidal stability, and rheological behaviour in order to increase the amount of resveratrol encapsulated using concentrated double W_1_/O/W_2_ emulsions.

Regarding the stability of the studied yoghurts (Figure 3), it can be seen that all yoghurts present a large stability with TSI values of less than 6 units in all cases, similar to the values obtained for emulsions with an internal phase ratio of 20/80, the ones with a larger stability in the present study.

Moreover, it can be appreciated that the control yoghurt is the most unstable, obtaining the highest TSI values. When emulsions were added to yoghurts, a greater yoghurt stability was observed with lower TSI values compared to the values registered for plain yoghurt.

Even though the emulsions with an internal ratio of 30/70 have a lower stability, their addition to the yoghurt seems to have a larger positive effect on yoghurt stability, especially in the case where RSV was encapsulated in it. A similar trend has been described in a previous study, where small amounts of a vesicular system containing resveratrol and other biocompounds were added to a yoghurt [15]. Moreover, these results also agree with those obtained in previous studies [56,57] in which double emulsions were added to yoghurts producing an increase in their stability.

Backscattering (BS) profiles obtained in the stability measurements for the double emulsions stored at 4 °C of both series of experiments (with or without RSV and with a ratio of concentration 20/80 or 30/70) and for all yoghurts formulated (with or without emulsions and RSV encapsulated) were studied.

The results obtained from backscattering of the studied emulsions showed that the 30/70 emulsions (with and without RSV) presented a great heterogeneity in their backscattering profiles over time, with a slight coalescence in the middle of the sample which indicates a certain degree of instability, while the 20/80 emulsions did not show notable changes. In addition, in the lower part of the cell that contained 30/70 emulsions, a clarification process was observed, behaviour that does not occur in the 20/80 emulsions. It is also seen that a low migration of the drops occurs in both types of emulsions, which rise to the top of the cell due to their lower density (creaming).

Figure S2 shows the results obtained from backscattering of the studied yoghurts. If the control yoghurt is observed (Figure S2A), it can be noted that there was no sedimentation, clarification, or creaming phenomena. Only a small certain degree of coalescence was observed in the middle part of the cell, which could be also due to the union of certain fatty or protein agglomerates typical of yoghurt; this was also observed by a reduction in particle size (Figure 1). This small variation is less in the samples that contain emulsion, which agrees with the results obtained from the TSI (Figure S2B–E). No large differences are found between the presence or absence of RSV on the emulsions added to the yoghurts but the addition of emulsions with an internal ratio 30/70 offers a larger stability, presenting a lower difference in particle size in the middle of the cell.

It can be concluded that the stability of the emulsion is not always related to the stability of the final matrix system where the emulsions are added. Interactions between oil drops and food matrix compounds could produce unexpected behaviour.

### 2.3. Rheology

#### 2.3.1. Flow Curves

**Figure 4:**
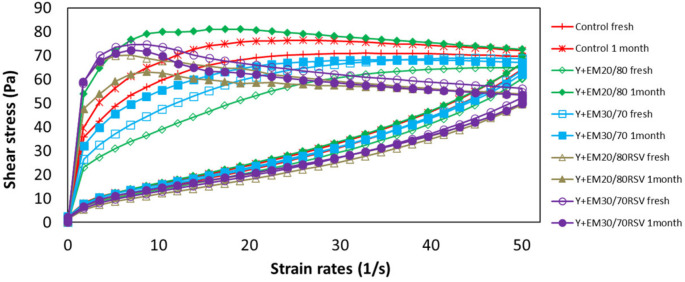
Upward and downward flow curves for both fresh yoghurts (with or without RSV) and those stored in the fridge for one month. The ten upper lines correspond to upward flow curves and the ten lower lines correspond to downward flow curves.

According to the shape of the upward flow curve, both the control yoghurt and the yoghurts with emulsion without RSV showed a clearly pseudoplastic behaviour, as can be seen in Figure 4. This behaviour has been previously described in yoghurts [58,59,60].

The samples containing RSV showed a behaviour that can be considered pseudoplastic in the initial part of the curve. However, at shear rates above about 10 s^−1^ the shear stress values tended to decrease. This behaviour may be related to the existence of areas within the yoghurt with different levels of deformation (shear-banding phenomena), which causes non-homogeneous velocity gradients during the viscosimetric flow of the samples. This heterogeneous flow of yoghurts is caused by the presence of RSV in the samples and has been previously described in highly concentrated emulsions and nanoemulsions [61,62].

In general, in all cases, the yoghurts showed a similar behaviour when fresh or after one month of storage, although small differences were observed between the different types of yoghurt. Specifically, the stress values reached at the beginning of the test were higher in the sample containing the emulsion without RSV 20/80 than in the 30/70 emulsion and higher in the latter than in the control, while after storage, the highest stress values were achieved in the yoghurt containing the emulsion without RSV 30/70, followed by the control and by the sample with the emulsion without RSV 20/80. This indicates slight changes in the stability of the samples. In the case of yoghurts containing RSV, the behaviour was similar fresh and after storage, which reflected a greater stability than the rest of the samples.

Table S2 show the results of the fitting to a Herschel–Bulkley model. It is observed that, as indicated by the r^2^ values, the control samples, as well as those containing the emulsion without RSV, showed a good fit to the model, while the yoghurts without RSV showed a much worse fit, which is due to the shear-banding phenomena commented above. In all cases, the yield strength (τ_0_) showed negative values. This negative value indicates that the yoghurt begins to flow with low stress values. The values of the behaviour index (*n*) indicate a clearly pseudoplastic behaviour of all the yoghurts (*n* < 1). Kashaninejad and Razavi [63] indicated that a higher content of milk fat favours a non-Newtonian behaviour. However, in the present work, apart from the fat globules or oil drops, an additional resistance to the fluid to flow can be found, due to the presence of RSV molecules which could increase repulsion between oil drops.

If the results obtained fresh and after one month in storage are compared, the reduction in the values of *n* in the control sample is remarkable, as well as in the yoghurt containing an emulsion without RSV 20/80, which indicates an increase in the pseudoplastic character of these samples with time.

In the study of the viscosity curves, it was observed that the behaviour was similar in all cases, that is, the viscosity decreased with increasing deformation speed, which again corroborates the pseudoplastic character of the analysed samples. This indicates that, when applying a higher deformation speed on the sample, a breakage of the structure occurs in such a way that this destabilisation leads to a decrease in viscosity [31].

In general, storage does not affect viscosity values. Only in the case of yoghurt with an emulsion without RSV 20/80, a slight increase in the initial values of the viscosity curve can be observed in the sample stored for one month at 4 °C, which is responsible for the differences observed on the flow curve measurements. This is in good agreement with results found by Toczek et al. [64], who reported that samples with a higher concentration of milk fat revealed higher values of viscosity.

Table S3 shows the thixotropy results of fresh yoghurts and those that were in the fridge for 1 month. All the values are in the same order of magnitude and there are no notable changes in thixotropy after storage time. Again, the values found in this work are in the range of those described previously in the literature regarding the thixotropic character of yoghurts [65,66].

#### 2.3.2. Frequency Sweep

In the analysis of the frequency sweep results of the different fresh elaborated yoghurts, it was observed without exceptions, that the elastic modulus (G’) presented higher values throughout the sweep than the viscous modulus (G’’), which indicates the predominantly elastic character of the samples, in accordance with previous results [58]. It was observed that, except for the G’’ values of the control sample and of yoghurt with an emulsion without RSV 20/80, the elastic and viscous moduli showed a clear dependence on frequency, increasing slightly as the angular velocity increases. This dependence on frequency values can be due to the fact that the emulsion 20/80 contained a slightly higher amount of fat in comparison with the rest of the samples and, as it has been reported, the fat content affects the elastic (G’) and storage (G’’) moduli behaviour of emulsions based on milk fat.

In the results of the yoghurts that were stored for 1 month at 4 °C, it was observed that, as with fresh yoghurts, G’ predominated over G’’ throughout the entire sweep. Similarly, the G’ and G’’ moduli showed a frequency dependence except for the viscous modulus values for the control sample and the yoghurt with emulsion without RSV 20/80. In general, the elastic and viscous modulus values are similar at the beginning of the test (fresh samples) and after storage. However, it should be noted that in the case of samples containing an emulsion without RSV, the values of G’ and G’’ increased slightly after being kept for a month at 4 °C, which indicates a lower stability of these samples, phenomenon that was observed when analysing the stability of samples containing RSV, since it was detected that RSV increased the stability of the colloidal system.

### 2.4. Texturometry

**Figure 5:**
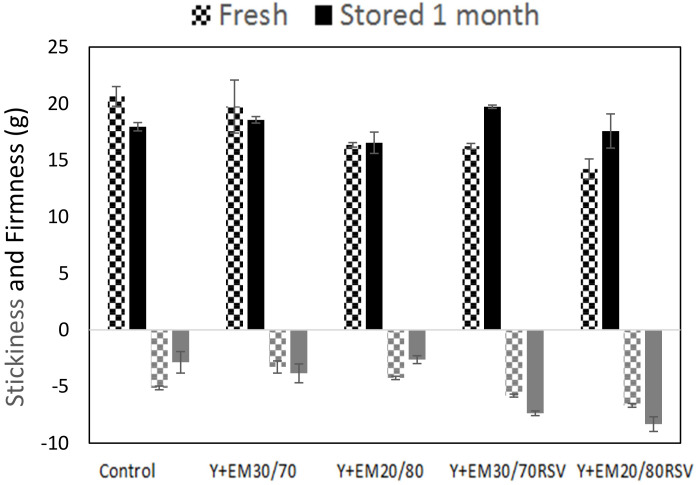
Firmness (above the *X* axis, in black colour) and stickiness (below the *X* axis, in grey colour) of the different types of yoghurts made comparing those that were fresh with those that were stored for 1 month at 4 °C.

Figure 5 shows the firmness and stickiness results obtained from the textural analyses, both for fresh yoghurts and for those that were stored for 1 month in a fridge. At time zero, the control yoghurt showed the highest firmness values, followed by the sample with an emulsion without RSV 30/70; the samples with an emulsion without RSV 20/80 and with an emulsion with RSV 30/70 showed similar values. The sample with an emulsion with RSV 20/80 presented the lowest firmness values. After storage, the firmness values decreased in the control sample and in the yoghurt with an emulsion without RSV 30/70, whereas the firmness remained more or less constant in the sample with an emulsion without RSV 20/80 and increased in the samples with an emulsion with RSV. On the contrary, previous studies [15] described that the encapsulation of RSV in niosomes, which were subsequently introduced into yoghurt, did not produce notable differences in terms of firmness when compared to the control. Likewise, in a study on the fortification of yoghurt using niosomes where iron is encapsulated [67], it was found that the firmness of yoghurt is increased by introducing niosomes with iron inside it, indicating that the colloidal system added to yoghurt has a great influence on its firmness. As a general trend it can be observed that samples with a larger fat content, those in which double emulsions formulated with an internal ratio of 20/80 were added, are the ones that presented a lower firmness value. Nevertheless, it should be considered that these were not significant differences, which is in accordance with the results reported by Toczek et al. [64]. These authors indicated that the effect of milk fat concentration with regards to stickiness and firmness was notable only for samples with a completely different composition.

Regarding the stickiness of fresh yoghurts (Figure 5), it can be seen that when adding an emulsion without RSV to the yoghurt, there is a decrease in stickiness, reaching the lowest values. However, when an emulsion containing RSV is added to the control yoghurt, an increase in stickiness occurs, obtaining the maximum values. This indicates that the presence of RSV increases the stickiness of the samples, probably due to the unions for this molecule located at the O/W interface that could interact with the proteins and fatty acids contained in the yoghurt, as it was also observed on the particle distributions measurements. After a month at 4 °C, it is observed that in the control sample the stickiness is reduced, while in the yoghurts with RSV it is increased. In the samples with an emulsion without RSV, in the case of the 30/70 emulsion, the stickiness increases and in the 20/80 emulsion it decreases.

Therefore, the presence of RSV is decisive in the yoghurt stickiness while firmness is more related to the yoghurt fat content.

### 2.5. Colorimetry

At time zero, in all the parameters (L*, a*, and b*), in general, similar values are observed; only in the case of the parameter a* are small differences between samples detected (Table S4 of Supplementary Materials). The same happens in the case of yoghurts after a month of storage. The high values of L* are noteworthy, which are mainly caused by the reflection of light by fat globules, calcium caseinate, and calcium phosphate colloidal particles contained in the milk [63]. This L* value typical of dairy products is in the same order of magnitude as those recently described in a work on frozen yoghurts [68] and in a study on fortified yoghurts [69].Therefore, neither the presence of the emulsion nor that of RSV notably affect the colour of the sample. However, if a colour variation (ΔE) is observed with the storage time, it can be seen that in all cases there is a difference in total colour change, the maximum difference happens in the yoghurt with emulsion with RSV 20/80 and the smallest difference in the control yoghurt. This indicates that the storage time hardly affects the colour of the control sample, on the contrary, in the yoghurt with an emulsion with RSV 20/80, a colour change is observed with time.

As a general trend, it can be observed that the presence of a higher fat content, as is the case of yoghurts with emulsions on them, causes a larger ΔE than in the control yoghurt, where the fat content is close to 10 times less in Table 1. However, no significant differences are observed on the L*, a*, and b* individual values (Table S4 from Supplementary Materials), indicating that the presence of fat does not affect the colour parameter of the prepared yoghurts.

### 2.6. Visual Inspection

The presence of double emulsions (W_1_/O/W_2_) in those yoghurts that contained them was confirmed since the water inner droplets (W_1_) immersed within the oil drops (O) were appreciably dispersed in the external aqueous matrix.

**Figure 6:**
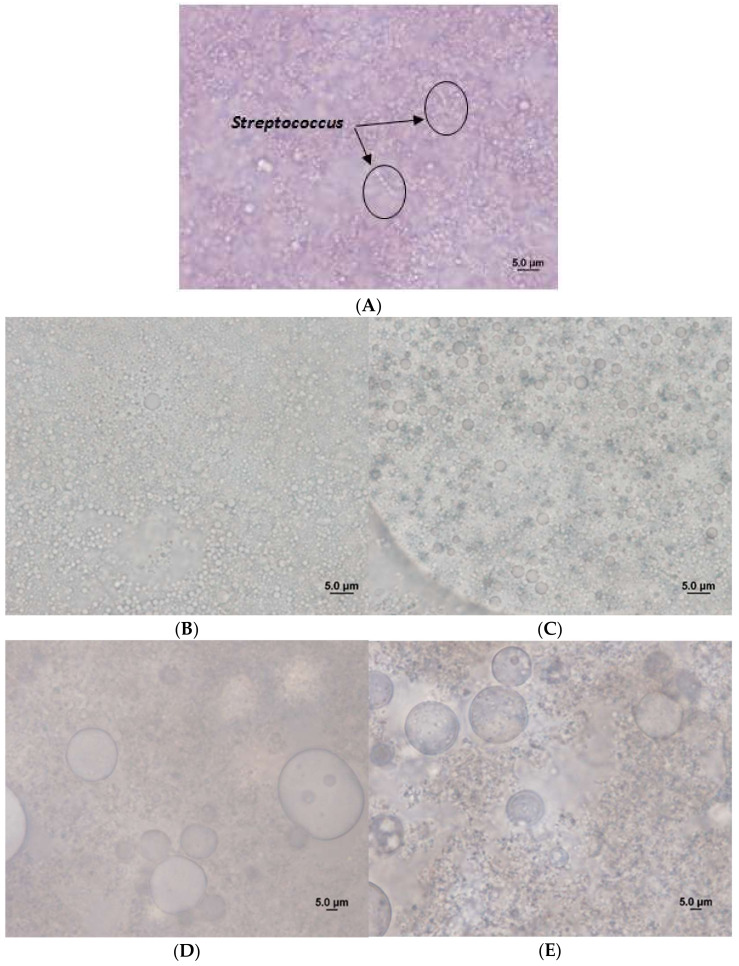
Micrographs corresponding to the different processed types of fresh yoghurt: control yoghurt (**A**); yoghurt with emulsion without RSV 30/70 (**B**); yoghurt with emulsion without RSV 20/80 (**C**); yoghurt with emulsion with RSV 30/70 (**D**); yoghurt with emulsion with RSV 20/80 (**E**).

Figure 6A shows a dense and compact structure typical of a yoghurt with the absence of emulsions and RSV. All samples showed the presence of lactic acid bacteria of the genus *Lactobacillus* or *Streptococcus* (the latter can be seen in Figure 6A, with the classic chain of *coccus* of the genus). In the rest of the micrographs of Figure 6B–E, it is clearly seen that the addition of emulsions to the yoghurt produced a modification of its structure, with the obvious presence of drops of emulsions of greater or lesser size. If the yoghurt with an emulsion without RSV 30/70 (Figure 6B) is compared with that of 20/80 (Figure 6C), there are no notable differences in droplet size, the droplets being slightly larger in the case of an emulsion with internal ratio of 20/80.

The samples containing RSV (Figure 6D,E) did show the presence of a number of larger drops indicating the presence of oil droplet coalescence, which seems to be promoted by the presence of RSV. Even stability values did not present significant changes on droplet size during the one-month storage, indicating that this large size observed was local and will not promote later coalescence during stability.

This coalescence it is even larger for emulsions with a 30/70 internal ratio, where the presence of larger drops is detected.

However, due to the large stability observed for all yoghurts, it seems that the presence of these large drops does not affect the average particle size of the final yo-ghurt. Moreover, due to the large viscosity of the yoghurt matrix, compared to the aqueous external phase, no creaming phenomenon promoted by the migration of the large oil drops to the surface was observed during the one-month study.

### 2.7. Antioxidant Activity

The antioxidant capacity of five different types of yoghurts was analysed: (i) plain yoghurt, (ii) yoghurt enriched with double emulsions (internal phase ratio: 20/80) and an RSV concentration of 0.5 mg/kg, (iii) yoghurt enriched with double emulsions (internal phase ratio: 30/70) and an RSV concentration of 0.8 mg/kg, (iv) yoghurt containing free RSV (0.5 mg/kg), (v) yoghurt containing free RSV (0.8 mg/kg). Results are presented in Table 2.

Plain yoghurts without resveratrol added showed an antioxidant capacity of 31 ± 0.7%, similar values to those obtained by Ahmed et al. (2021) [70]. Yoghurts loaded with non-encapsulated RSV and with emulsions containing 0.5 mg/kg of RSV showed values similar to the ones obtained with the plain yoghurt. However, a significant increase was observed for yoghurts containing an RSV concentration of 0.8 mg/kg, around 35 ± 0.9%.

After one month of storage at 4 °C the antioxidant capacity of yoghurts was measured. Yoghurts with RSV encapsulated presented a 3% larger inhibition than those containing free RSV. A significant reduction was observed for yoghurt with free RSV at the largest concentration studied (0.8 mg/kg).

Based on these results, it can be concluded that the emulsions effectively encapsulate and protect RSV from its oxidation by external agents. However, due to the antioxidant capacity of the plain yoghurt, the addition of at least 0.8 mg/kg will be required to observe significant differences on the antioxidant capacity of the final enriched food.

## 3. Materials and Methods

### 3.1. Materials

*Trans*-resveratrol (C_14_H_12_O_3_), absolute ethanol, and Tween 20 (HLB 16.7) were supplied by Sigma–Aldrich (St. Louis, MO, USA). The neutral oil Miglyol ^®^ 812 with a density of 945 kg/m^3^ at 20 °C was purchased from Sasol GmbH. Regarding emulsifiers, it was necessary to acquire polyglycerol polyricinoleate (PGPR, HLB 3.0) from supplier Brenntag AG and Tween 20 (HLB 16.7) from Sigma–Aldrich. NaCl was obtained from Panreac. For the preparation of internal and external aqueous phases (W_1_ and W_2_) we used deionized water (MilliporeElix 5, Merck, Kenilworth, NJ, USA).

White label plain yoghurts (Alimerka, Gijon, Spain) were used for the preparation of the 5 types of yoghurts. It was also necessary to acquire skimmed milk powder from the Mercadona supermarket brand (Tavernes Blanques, Spain), as well as whole milk (Cremosita, Valdemoro, Spain).

### 3.2. Methods

Five types of yoghurts were prepared: (i) blank (without RSV and without emulsion), (ii) yoghurt with a 30/70 double emulsion without RSV (Y+EM30/70), (iii) yoghurt with a 20/80 double emulsion without RSV (Y+EM20/80), (iv) yoghurt with a 30/70 double emulsion with RSV (Y+EM30/70RSV) and (v) yoghurt with a 20/80 double emulsion with RSV (Y+EM20/80RSV).

For the preparation of the primary W_1_/O emulsions, two different internal ratios of internal aqueous phase in the oil phase (also represented as W_1_/O) were used: 20/80 and 30/70. The external ratio of the primary emulsion (W_1_/O) in the external aqueous phase W_2_ (also represented as W_1_O/W_2_) was the same for all the concentrated double W_1_/O/W_2_ emulsions formulated: 80/20.

The double emulsions and the different involved phases were prepared following the method described in previous works [53].

#### 3.2.1. Water-in-Oil (W_1_/O) Inner Emulsion Preparation

To ensure W_1_ droplet stability in all double emulsions formulated, NaCl was added with a concentration in the inner aqueous phase of 0.1 M. Thus, the attractive force between water droplets was lowered with the presence of electrolytes [71,72,73].

It was necessary to prepare a 20% ethanol (*v*/*v*) solution containing 50 mg/L of *trans*-resveratrol as the W_1_ phase for the emulsions because this natural polyphenol is highly soluble in ethanol but barely soluble in water. It has been proved that its solubility in alcohol increases as the carbon number of the alcohol decreases [74].

As continuous phase of the inner emulsion (oily phase), we used a solution of Miglyol 812 oil with the hydrophobic surfactant PGPR dissolved by magnetic stirring for 5 min. This surfactant was chosen because it is commonly used in food formulations and is highly effective at stabilizing W_1_/O emulsions [73,75,76].

The PGPR/internal aqueous phase ratio was kept constant to ensure that the surface of the dispersed droplets (W_1_) was completely covered by the PGPR. The PGPR concentration used for a simple W_1_/O emulsion ratio of 20/80 was 20% (*w*/*w*) of PGPR while 40% (*w*/*w*) was used for a simple W_1_/O emulsion prepared with the 30/70 ratio, to avoid the instability that occurs when the same concentration of surfactant for higher internal phase proportions is maintained [31].

Two W_1_/O volumetric ratios were studied, 20/80 and 30/70. The first step of emulsification (W_1_ in O) was carried out by employing a SilentCruser M Homogenizer (Heidolph, Schwabach, Germany) using a 6 mm dispersing tool. The high-shear mixing was performed at 15,000 rpm for 5 min. 

#### 3.2.2. Water-in-Oil-in-Water (W_1_/O/W_2_) Double Emulsions Preparation

The proportion of W_1_/O primary emulsion dispersed into the external aqueous phase (W_2_) was 80% of primary emulsion into 20% of W_2_, therefore the ratio of W_1_/O/ W_2_ was 80/20.

The external aqueous phase was prepared using a 2% (*w*/*v*) solution of Tween 20 as stabiliser of the external aqueous phase and 0.1 M NaCl (magnetic stirring for 30 min). The sodium chloride equilibrated the osmotic pressure between aqueous phases.

The second step of emulsification was carried out by mixing the W_1_/O and W_2_ phases with the homogeniser used in the first emulsification stage but at 5000 rpm for 2 min. The speed and time of the second step of emulsification were lower to avoid breaking the initial emulsion (W_1_/O) obtained in the first step.

The final RSV concentration on the highly concentrated emulsions prepared were 8.4 and 12.4 mg RSV/kg of double emulsion, for double emulsions with ratios 20/80 and 30/70, respectively.

#### 3.2.3. Yoghurts Preparation

Yoghurts were prepared by adding to a litre of whole milk, 28.45 g of milk powder and 68.58 g of plain yoghurt. The mixture was stirred and homogenised for 10 min at 1000 rpm at room temperature. Then, the corresponding emulsion formulated was added in the following proportion: 5 g (w/w) of double emulsion (20/80 or 30/70 emulsion with or without RSV) and 75 g of the aforementioned mixture of milk and yoghurt. No emulsion was added to the blank, so it consisted in 80 g of the mixture.

Yoghurts with RSV contained a final concentration of 0.5 and 0.8 mg/kg, for yoghurts enriched with 20/80 and 30/70 emulsions, respectively

Once the mixture was prepared, it was maintained for 12 h at 30 °C in an oven and then placed in a fridge at 4 °C for 3 h.

#### 3.2.4. Yoghurt Characterisation

All yoghurts prepared were characterised in terms of droplet size, morphology, rheology, texturometry colour, stability, and antioxidant capacity. The measurements were carried out in triplicate. The addition of emulsions to the food matrix as yoghurt in the present case, could produce some colloidal interactions between oil droplets, stabilisers, and proteins or fat globules present in the yoghurt. In that sense and in order to evaluate the influence of the presence of the double emulsion on the prepared yoghurt, a physical characterisation was made in fresh conditions and after one month of storage at 4 °C, as it is the typical temperature used for yoghurt storage.

Moreover, in order to have a deeper knowledge and easily understand the potential changes that prepared yoghurts could have over one month, the storage emulsion stability was also analysed over one month of storage at 4 °C.

##### Droplet Size Distribution

A Mastersizer S long bench apparatus (Malvern Instruments) based on a laser light scattering technique was used to obtain particle size distributions. 

To measure the particle size of the yoghurts, samples were prepared in solution according to the protocol described by other authors [77]: 0.2 g of the sample with yoghurt, 0.1% of the tensioactive sodium dodecyl sulfate (SDS) and 150 mL of distilled water. Once these components were mixed, they were shaken to achieve homogenisation of the samples, to be subsequently analysed in the Mastersizer.

##### Colloidal Stability

A Turbiscan apparatus (Formulaction, France) was used to study the emulsion stability by measuring the backscattering and transmission profiles, without requiring a dilution. The Turbiscan monitored the backscattered (BS) and transmitted (TS) light of the double emulsions and of the yoghurts as a function of time and cell height during the storage month at 4 °C. The Turbiscan records the data of every 40 μm of the cell and represents the BS and TS in percentage relative to the standards (suspensions of monodisperse spheres of latex in silicone oil) as a function of the sample height (in mm). These profiles provide useful information about changes in droplet size distribution, appearance of a creaming layer or clarification front with time.

The Turbiscan Stability Index (TSI) is defined by Equation (1) and is calculated as the sum of all the size and/or concentration variations detected in the samples:

(1) $$TSI=\underset{i}{\sum }\frac{\sum_{i}\left|scan_{i}-scan_{i-1}\right|}{H}$$

where *H* is the total height of the cell at the i interval time. Therefore, the lower the TSI value, the greater the stability of the analysed sample.

##### Rheology

A Haake MARS II rotational rheometer (ThermoFisher Scientific, Waltham, MA, USA) with a plate/plate measuring system (PP60Ti with a gap of 1 mm) was used to carry out the rheological tests. The temperature of analysis (5 °C) was controlled with a Peltier unit. Before each measurement, it was necessary to wait at least 15 min to reach the temperature of 5 °C and to relax the stresses induced during sample loading. 

For the rheological characterization, two different types of tests were carried out: flow curve and frequency sweep. 

Flow curve was conducted as follows, an upward flow curve was made increasing the shear rate from 0.001 to 50 s^−1^ in 180 s, once 50 s^−1^ was reached, this shear rate was maintained for 10 s and, finally, a downward flow curve was made by reducing the shear rate from 50 to 0.001 s^−1^ in 180 s. In this way, the thixotropic character of the sample can be evaluated by obtaining the hysteresis area between both curves. 

The data obtained in the upward flow curves have been adjusted to the Herschel–Bulkley Equation (2), as it is the rheological model most widely used to describe real non-Newtonian fluids behaviour.

(2) $$\tau =\tau_{0}+K\dot{\gamma }{\;}^{n}$$

where *τ* is the shear stress (in Pa), *τ*_0_ is the yield point (in Pa), $\dot{\gamma }$ is the shear rate (in s^−1^), K is the consistency coefficient (in Pa s^-1^) and n is the flow behaviour index (dimensionless).

Frequency sweep was carried out from 0.1 to 500 rad/s at a constant shear stress of 5 Pa, measuring the elastic and viscous moduli.

##### Texturometry

A TA.XT.plus texture analyser (Stable Micro Systems, Godalming, UK) was used to perform the texturometry tests. The penetration test was carried out with a load cell of 5000 g and a 0.5-inch diameter probe. The recorded data were force versus time. The maximum force was considered as a measurement of firmness. However, in the opposite side, the maximum negative force was considered as a measurement of cohesiveness. 

##### Colorimetry

To carry out the analysis, an SV 100 spectrocolorimeter (Lovibond) was used, employing a quartz cuvette with a 10 mm light path. The colour was measured in terms of CIELAB parameters L* (whiteness or brightness), a* (redness or greenness), and b* (yellowness or blueness) [78].

To compare the colour change of the fresh samples with those close to their expiration date (1 month), the ∆E (total colour change) was calculated as follows [79]:

(3) $$∆E=\sqrt{{\left(∆L^{*}\right)}^{2}+{\left(∆a^{*}\right)}^{2}+{\left(∆b^{*}\right)}^{2}}$$

#### Visual Inspection

The particle size obtained with the Mastersizer device was confirmed by obtaining micrographs of the yoghurts. This visual inspection allowed us to study the change in the structure after adding the emulsion to the yoghurt. An Olympus BX50 light microscope was used to obtain the micrographs of the formulated emulsions. It was used with a 10–100× magnification using UV–vis. Moreover, the visual inspection allowed us to gain a deeper knowledge regarding the sample particle size, providing information about the possible presence of aggregates instead of individual particle/droplets. 

#### Antioxidant Activity

The antioxidant activity of yoghurts was determined by measuring the DPPH (2,2-diphenyl-1-picrylhydrazyl) free radical scavenging activity according to Moussi et al. [80]. Absorbance of these mixtures were measured, by UV–vis spectrophotometer, at 517 nm.

The measurements were carried out in triplicate and the inhibition percentage was calculated according to Equation (4):

(4) $$Inhibition\;\left(\%\right)=\frac{A_{0}-A_{s}}{A_{s}}\cdot 100$$

where *A_0_* was the absorbance of the blank sample and *A_s_* was the absorbance of each yoghurt analysed.

#### Statistical Analysis

All data were expressed as the mean ± SD (standard deviation) of three independent experiments, and the statistical analysis of the data was carried out (ANOVA). Fisher’s test (*p* < 0.05) was used to calculate the least significance difference (LSD) using statistical software (Microsoft Excel 2010).

## 4. Conclusions

The feasibility of preparing enriched yoghurts with highly concentrated double emulsions (80% volume of W_1_/O in W_2_) containing RSV has been proven.

The formulation that presented larger stability was the one prepared with a simple W_1_/O ratio of 20/80.

The presence of the double emulsions in the fortified yoghurts reduced the yoghurt droplet size and produced an increase in its stability. The addition of RSV in the incorporated emulsions did not affect the yoghurt stability.

Rheological measurements showed that the presence of double emulsions did not alter yoghurt rheological properties. However, in the case of yoghurts with emulsion containing RSV, shear-banding phenomena were observed, indicating that the RSV could be located at the water/oil interface, offering additional resistance to the continuous phase to flow between oil drops. The yoghurts showed pseudoplastic and thixotropic characteristics and a predominant elastic behaviour.

Encapsulated RSV added to yoghurts offered a larger protection compared with free RSV, presenting a larger antioxidant inhibition after a one-month storage. Moreover, the inhibition was nearly nonaffected by storage, presenting maximum values of reduction of 3% after one month. An appreciable increase of the antioxidant capacity of the final functional yoghurt was observed, when the RSV concentration in the final fortified food arose to 0.8 mg/kg.

Yoghurts enriched with emulsions containing RSV showed physical properties such as size, colour, rheological behaviour, and textural properties, similar for fresh and one-month-old samples, and similar to those observed on control yoghurts. Hence, double emulsions could be considered as suitable carriers for further industrial and commercial purposes.

Since most health benefits of the dietary polyphenols are exerted through the intestinal tract to the colon, future research should be aimed to study the interactions between RSV contained in enriched yoghurts and gut microbiota, in order to increase the knowledge on the mechanisms of the beneficial effects of this nutraceutical on the host’s health.

## Figures and Tables

**Table 1:** Colour difference (ΔE) observed when relating each fresh sample with its counterpart after 1 month of storage in a fridge.

	Control	Y+EM20/80	Y+EM30/70	Y+EM20/80RSV	Y+EM30/70RSV
ΔE	0.081 ± 0.05	0.281 ± 0.02	0.420 ± 0.08	1.105 ± 0.08	0.350 ± 0.11

**Table 2:** Antioxidant capacity of samples prepared fresh and after one month of storage: plain yoghurts, yoghurts containing free RSV (0.5 mg/kg and 0.8 mg/kg) and yoghurts containing emulsions with RSV (0.5 mg/kg and 0.8 mg/kg).

Type of Yoghurt	Antioxidant Capacity (Fresh)	Antioxidant Capacity (After One Month)
Plain yoghurt	31 ± 0.7% ^a,x^	31 ± 0.9% ^h^,^x^
Yoghurts enriched with double emulsions (20/80) with RSV (0.5 mg/kg)	32 ± 0.6% ^b,x^	32 ± 1.0% ^h^,^x^
Yoghurts enriched with double emulsions (30/70) with RSV (0.8 mg/kg)	35 ± 0.9% ^c,x^	36 ± 1.1% ^i,x^
Yoghurts containing free RSV (0.5 mg/kg)	30 ± 0.6% ^d,x^	30 ± 0.8% ^h,x^
Yoghurts containing free RSV (0.8 mg/kg)	35 ± 0.9% ^c,x^	31 ± 0.8% ^h,y^

^a,b,c,d^: significant differences among fresh samples; ^h,i^: significant differences among one-month-old samples; ^x,y.^ significant difference between fresh and one-month-old sample

## Supplementary Materials

The following are available online at https://www.mdpi.com/article/10.3390/ijms23010085/s1.
