# Supplementary material for: Addition of Trans-Resveratrol-Loaded Highly Concentrated Double Emulsion to Yoghurts: Effect on Physicochemical Properties
Source: Int J Mol Sci. 2021 Dec 22;23(1):85. doi: 10.3390/ijms23010085 (PMC8744663; doi:10.3390/ijms23010085)
Supplement: Supplementary file 1 [file ijms-23-00085-s001.zip › ijms-1497942-supplementary.pdf]

Addition of Trans resveratrol loaded highly concentrate double emulsion to yoghurts: effect on physicochemical properties

Rocío DÍAZ-RUIZ [a,b] (uo219049@uniovi.es), Manuel Ramón FERNÁNDEZ [a] (UO245369@uniovi.es), Amanda LACA[a] (lacaamanda@uniovi.es), Marta Sánchez[a] (UO209096@uniovi.es), María MATOS [a,b] (matosmaria@uniovi.es), Gemma GUTIÉRREZ [a,b,1](gutierrezgemma@uniovi.es)

[a] Department of Chemical and Environmental Engineering, University of Oviedo, Julián Clavería 8, 33006 Oviedo, Spain.

[b] Instituto Universitario de Biotecnología de Asturias, University of Oviedo, 33006, Spain

Corresponding autor: Gemma Gutiérrez; Tel.: +34 985103509; Fax: +34 985103434

---

[1]Corresponding author email: gutierrezgemma@uniovi.es

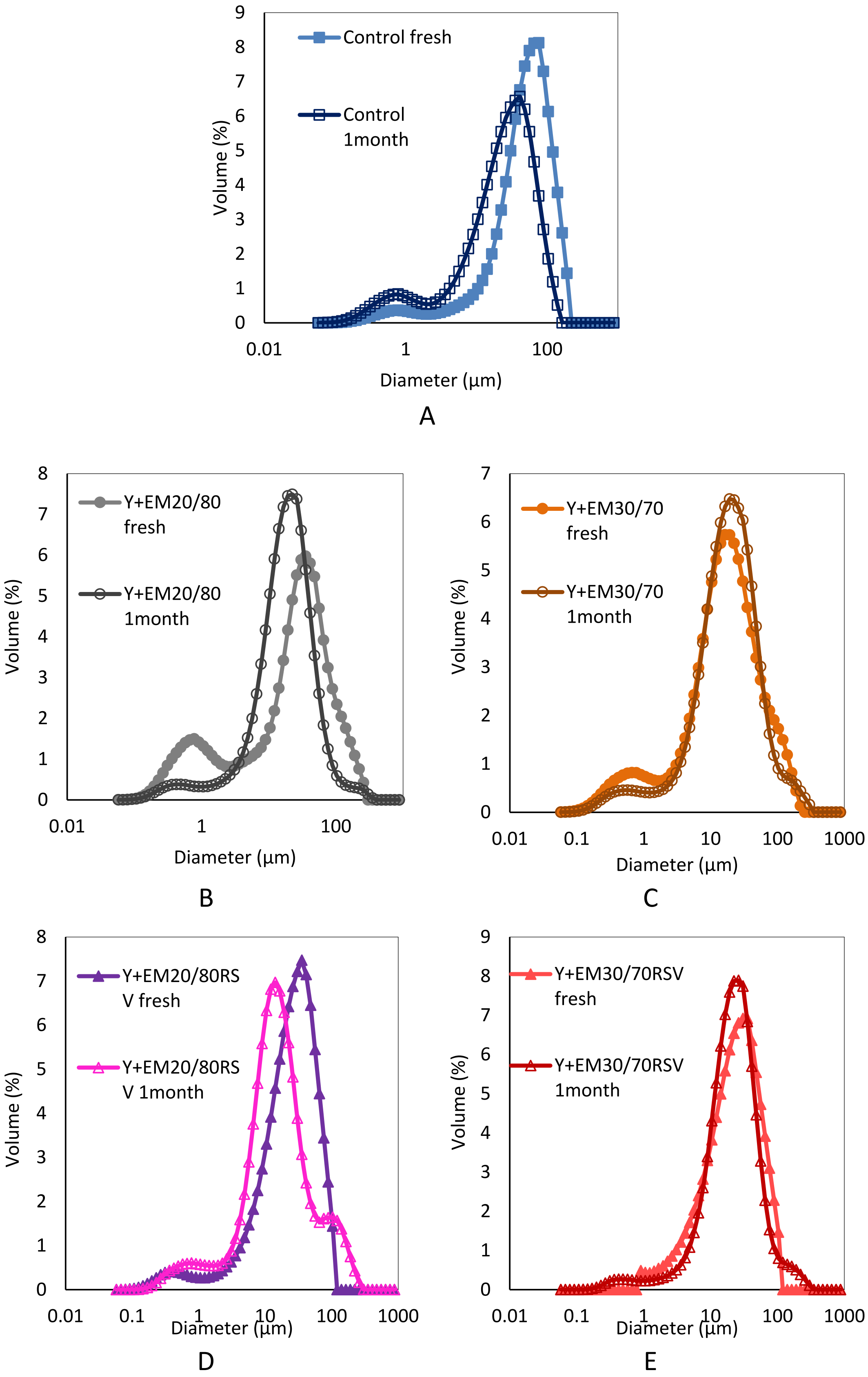

Figure S1. Droplet size distribution of the different elaborated yoghurts, comparing each fresh yoghurt with its monthly counterpart.

Table S1. Mean diameters, D(v,0,5) and $D_{[4,3]}$, of the different samples prepared: control yoghurt and yoghurts with addition of double emulsions (EM20/80 and EM30/70) without and with RSV

	**Fresh**		**After storing 1 month at 4˚C**	
**Type of yoghurt**	**D(v,0.5)**	**$D_{[4,3]}$**	**D(v,0.5)**	**$D_{[4,3]}$**
Control	48.57	56.06	23.43	29.73
Y+EM20/80	24.53	36.21	17.25	24.27
Y+EM30/70	15.85	26.06	17.27	26.79
Y+EM20/80 RSV	23.74	28.49	20.16	27.25
Y+EM30/70 RSV	21.50	26.98	21.92	27.15

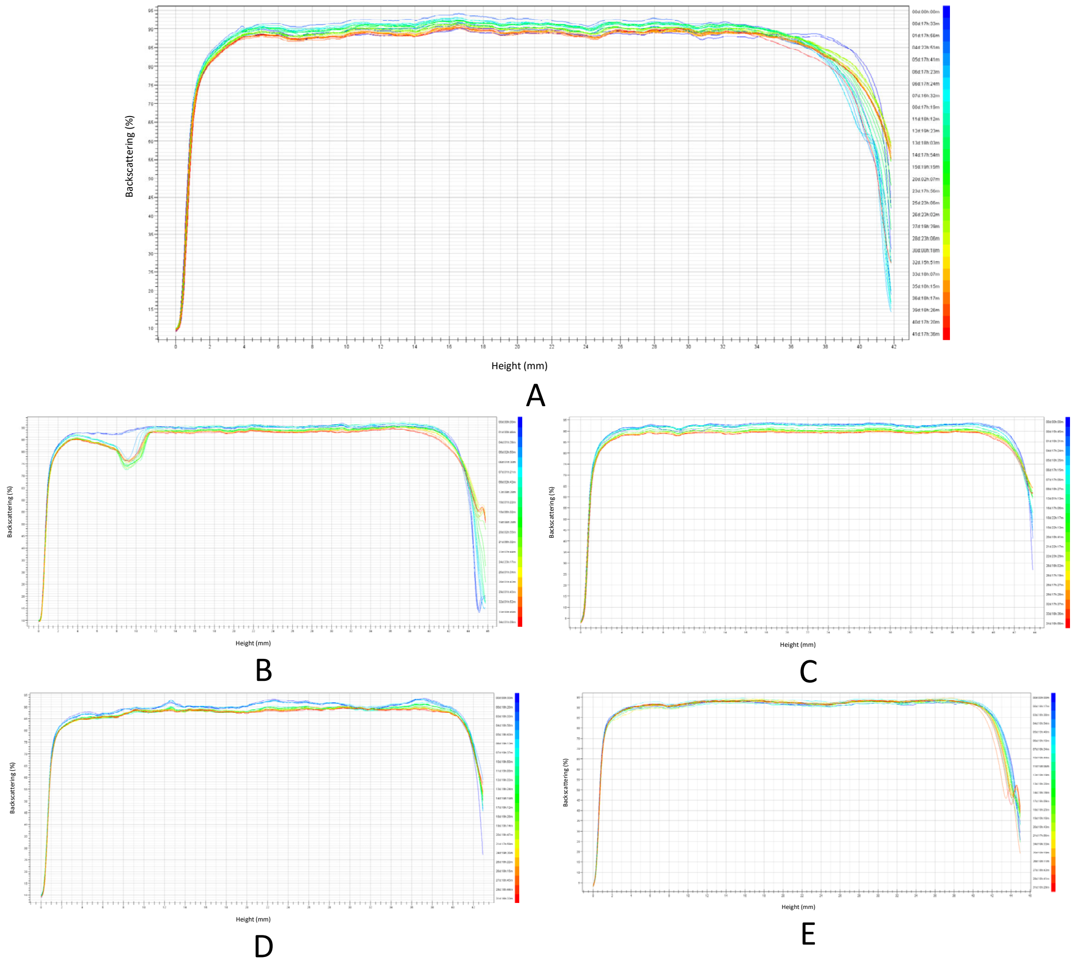

Figure S2. Backscattering profiles of the different processed types of yoghurt: Control yoghurt (A); Yoghurt with emulsion without RSV 20:80 (B); Yoghurt with emulsion without RSV 30:70 (C); Yoghurt with emulsion with RSV 20:80 (D);Yoghurt with emulsion with RSV 30:70 (E)

Table S2. Results of the adjustment to the Herschel-Bulkley model of fresh and stored yoghurts for 1 month.

		τ_0 (Pa)	*K* (Pa s^n)	n (-)	r^2
Fresh	Control	-19.7 ± 0.01	58.5 ± 0.73	0.12 ± 0.001	0.95 ± 0.0005
	Y+EM20/80	-0.76 ± 2.9	20.5 ± 3.9	0.32 ± 0.04	0.98 ± 0.004
	Y+EM30/70	-4.68 ± 1.08	29.8 ± 3.63	0.25 ± 0.03	0.97 ± 0.01
	Y+EM20/80RSV	-1632 ± 158	1681 ±161	0.002±0.0004	0.57 ± 0.16
	Y+EM30/70RSV	-1595 ± 734	1647 ± 739	0.003±0.0013	0.561 ± 0.13
After one month of storage	Control	-58.4 ± 7.33	101.5 ± 1.1	0.075 ± 0.007	0.934 ± 0.02
	Y+EM20/80	-1441 ± 525	1499 ± 526	0.004 ± 0.001	0.86 ± 0.004
	Y+EM30/70	-6.29 ± 4.7	34.7 ± 10.8	0.21 ± 0.055	0.97 ± 0.02
	Y+EM20/80RSV	-1539 ± 366	1584 ± 374	0.003 ± 0.0003	0.795 ± 0.08
	Y+EM30/70RSV	-2131 ± 64	2181 ± 62	0.002 ± 0	0.63 ± 0.045

Table S3. Thixotropy values of yogurts at time zero (fresh) and after 1 month stored in a fridge.

	Thixotropy (Pa/s)	
Type of yoghurts	Fresh	After 1 month stored
Control	1746 ± 0	1835 ± 136
Y+EM20/80	1293 ± 0	2176 ± 6.4
Y+EM30/70	1443 ± 139	1606 ± 298
Y+EM20/80RSV	1847 ± 187	1571 ± 344
Y+EM30/70RSV	1915.5 ± 200	1735 ± 65

Table S4. Results obtained after the colorimetric analysis of yogurts at time zero (fresh) and after 1 month stored in a fridge

	Type of yoghurts				
	**Fresh**				
	Control	Y+EM20/80	Y+EM30/70	Y+EM20/80RSV	Y+EM30/70RSV
**L***	84.9 ± 0.9	85.0 ± 0.25	84.4 ± 0.97	84.5 ± 0.21	84.7 ± 0.7
**a***	0.20 ± 0.1	0.23 ± 0.06	0.28 ± 0.18	0.35 ± 0.07	0.2 ± 0.28
**b***	4.77± 0.4	4.87 ± 0.15	4.87 ± 0.15	4.85 ± 0.21	4.95 ± 0.07
	**Month**				
**L***	84.95 ± 0.07	84.95 ± 0.21	84.8 ± 0.66	85.7 ± 0.1	85 ± 1.27
**a***	0.25 ± 0.07	0.15 ± 0.21	0.28 ± 0.10	0.40 ± 0.14	0.3 ± 0.00
**b***	4.7 ± 0.14	4.60 ± 0.28	4.73 ± 0.10	4.80 ± 0.07	4.80 ± 0.14
